# Diagnosing new-onset asthma in a paediatric clinical trial setting in school-age children

**DOI:** 10.3389/falgy.2024.1418922

**Published:** 2024-07-15

**Authors:** Graham Roberts, Erkka Valovirta, Susanne Halken, Peter A. Eng, Mika J. Mäkelä, Karin C. Lødrup Carlsen, Roland Knecht, L. Pekka Malmberg

**Affiliations:** ^1^ The David Hide Asthma and Allergy Research Centre, St Mary’s Hospital, Newport, Isle of Wight, United Kingdom; ^2^ NIHR Biomedical Research Centre, University Hospital Southampton NHS Foundation Trust, Southampton, United Kingdom; ^3^ Faculty of Medicine, University of Southampton, Southampton, United Kingdom; ^4^ Terveystalo Allergy Clinic, Department of Lung Diseases and Clinical Immunology, University of Turku, Turku, Finland; ^5^ Hans Christian Andersen Children’s Hospital, Odense University Hospital, Odense, Denmark; ^6^ Section of Paediatric Pulmonology and Allergy, Children’s Hospital, Aarau, Switzerland; ^7^ Skin and Allergy Hospital, Helsinki University Central Hospital and University of Helsinki, Helsinki, Finland; ^8^ Department of Paediatrics, Oslo University Hospital, Oslo, Norway; ^9^ Faculty of Medicine, Institute of Clinical Medicine, University of Oslo, Oslo, Norway; ^10^ Doctors Surgery for Paediatrics, Bretten, Germany; ^11^ Unit of Clinical Physiology, Skin and Allergy Hospital, Helsinki University and Helsinki University Hospital, Helsinki, Finland

**Keywords:** allergen immunotherapy, children, clinical trials, diagnosis, new-onset asthma

## Abstract

Asthma is a common chronic disease in children. It is a dynamic condition—symptoms change over time, and the outcome of diagnostic tests can vary. Consequently, evaluating the onset of asthma at a single point in time, perhaps when patients are asymptomatic with limited impairment of the lung function, may result in false diagnostic conclusions. The absence of consistent gold-standard diagnostic criteria in children challenges the ability of any study to ascertain an effect of treatment on asthma prevention. A comprehensive review of the diagnostic criteria used for new-onset asthma in school-age children was conducted based on existing recommendations from published clinical guidance, alongside evidence from paediatric asthma prevention trials. Findings from the review were used to propose suggestions for diagnosing new-onset asthma in future asthma prevention trials. Despite an overall lack of consensus in the published clinical guidance, there are similarities between the various recommendations for diagnosing asthma in children, which typically involve assessing the variable symptoms and supplementing the medical history with objective measures of lung function. For future paediatric asthma prevention trials, we suggest that paediatric clinical trials should use a new-onset asthma definition that incorporates the concepts of “possible”, “probable” and “confirmed” asthma. “Possible” asthma would capture self-reported features of chronic symptoms and symptom relief with β_2_-agonist bronchodilator (suggesting reversibility). “Probable” asthma would include symptom chronicity, self-reported symptom relief with β_2_-agonist bronchodilator, and objective features of asthma (reversibility or bronchial hyper-responsiveness). A “confirmed” diagnosis would be made only if there is a positive response to controller therapy. These suggestions aim to improve the diagnosis of new-onset childhood asthma in clinical trials, which will be useful in the design and conduct of future paediatric asthma prevention trials.

## 1 Introduction

Asthma is a major public health concern affecting more than 250 million people worldwide (2019 data) (1), and it is one of the most common chronic diseases in children (2). The underlying pathophysiology is heterogeneous, although the Th2-high phenotype predominates in the paediatric age group (3). Often, allergic and/or eosinophilic airway inflammation is present, and symptoms can be triggered by allergens, infections, or irritants such as pollution (3). The prevalence of asthma is higher in males than in females during childhood, but the situation reverses after adolescence and moving into adulthood when the predominance is female (4, 5).

Asthma is a disease of variable airflow obstruction, and no gold-standard diagnostic test for asthma exists (6). In clinical practice, asthma is diagnosed by a history of respiratory symptoms, such as wheeze, shortness of breath, chest tightness, and cough, that vary over time and in intensity, with variable expiratory airflow limitation [according to the Global Initiative for Asthma (GINA)] (7, 8). However, symptoms of wheeze, shortness of breath, and cough, in school-age children, may also be caused by conditions other than asthma—for example, bacterial or viral respiratory tract infections, congenital heart disease, or cystic fibrosis (8). Such conditions need to be excluded before a diagnosis of asthma is made (8). Wheeze, in particular, is common in preschool-age children (typically associated with upper respiratory tract infections) (8), making it a challenge to diagnose asthma in older, school-age children who have a history of wheezing.

In children, asthma is associated with comorbidities, impaired quality of life, limitations of physical activities/school performance, and psychological effects (9, 10). Asthma is also an important contributing factor for emergency department visits and hospitalisations in children (10, 11). The chronic, persistent nature of asthma (12) emphasises the need for preventive strategies. One such approach is to treat the underlying allergy that seems to be associated with much of childhood asthma (13, 14). Allergen immunotherapy (AIT) has been shown to have a preventive long-term effect on the development of asthma symptoms, the use of asthma medication, and on bronchial reactivity (15, 16). A window of opportunity for preventing asthma may exist during infancy in the early stages of immune development (17), or in young children during the early stages of disease where the level of allergic sensitisation is low (18). A meta-analysis of AIT trials suggested that AIT has a short-term benefit in preventing asthma in patients with allergic rhinitis (AR), particularly if AIT has been initiated in childhood (19). Other approaches, such as targeting microbial diversity, may also be important in primary asthma prevention (17). Despite the clinical evidence for AIT in asthma, the European Academy of Allergy and Clinical Immunology (EAACI) guidelines emphasise the need for further confirmatory studies (16). The EAACI guidelines also highlight the urgent need to define and standardise optimal clinical diagnostic criteria for asthma that should be used in future clinical trials (16).

With this in mind, we set out to review the criteria used to define new-onset asthma in the context of a paediatric clinical trial. This project developed from the authors' involvement in the Grazax Asthma Prevention (GAP) trial and their scientific discussions on the appropriateness of the definition for new-onset asthma used in the GAP trial (20). Here, we examine the recommendations for diagnosing paediatric asthma in clinical practice as outlined in various clinical guidelines and consider the challenges of defining diagnostic criteria for new-onset asthma in paediatric asthma prevention trials. We also provide suggestions for improving the definition of new-onset asthma in school-age children in the context of clinical trials, for use in future studies.

## 2 Methods

**Figure 1:**
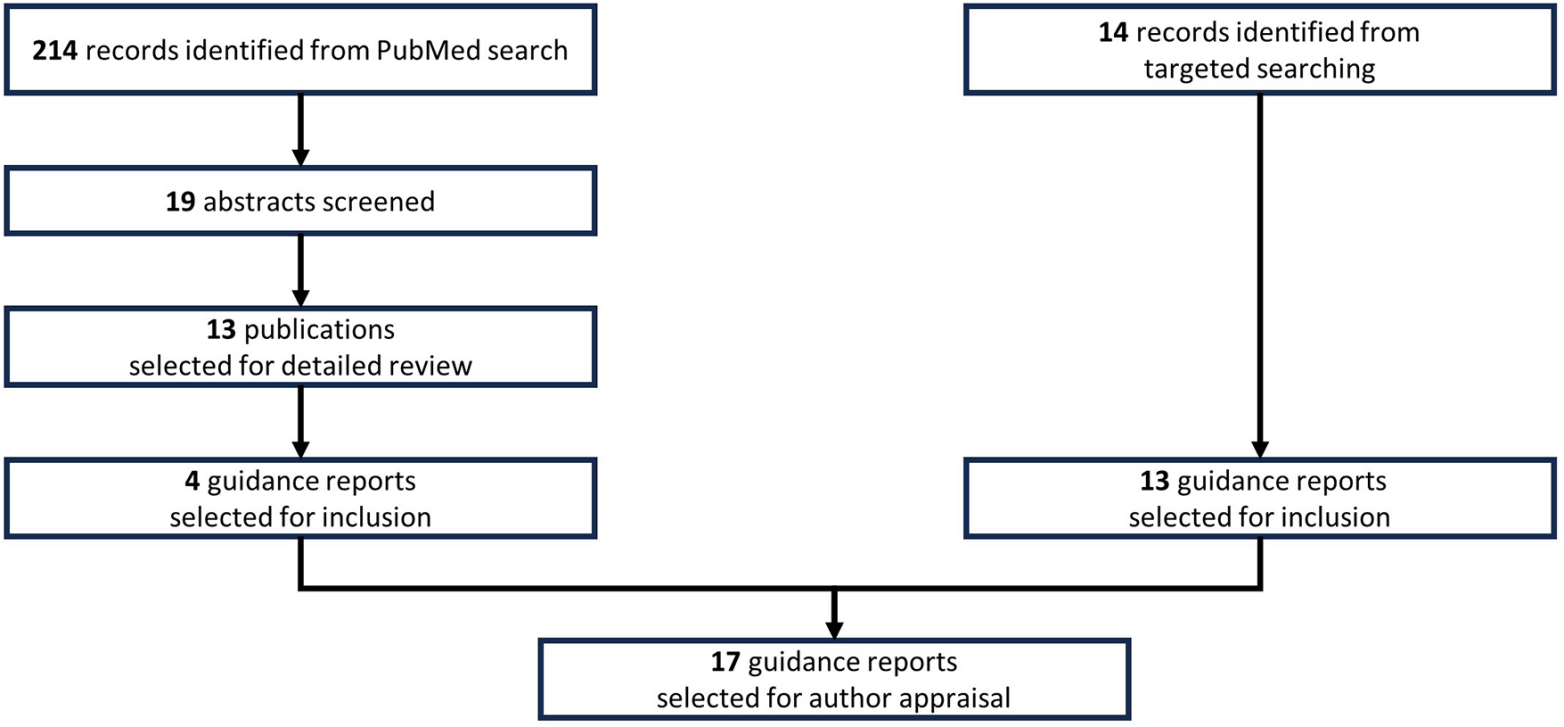
Literature searching conducted to identify clinical guidance reports containing recommendations on the diagnosis of asthma in school-age children.

Figure 1 outlines the literature searching that was undertaken to identify clinical guidance reports containing recommendations for the diagnosis of asthma in children. Firstly, a PubMed search was conducted (on 29 June 2023) using the search string: {[children(Title)] OR [childhood(Title)] OR [paediatric(Title)] OR [pediatric(Title)]} AND [asthma(Title)] AND {[guidelines(Title)] OR [guideline(Title)]}. The returned abstracts were screened for relevance (i.e., guidance for diagnosing asthma in children, written in English) and were excluded if they did not meet the required criteria. For selected abstracts, the full publication was evaluated for inclusion/exclusion using the same criteria. In addition, targeted literature searching of relevant international organisations for consensus reports and other guidance documents not captured by the PubMed search, was conducted using the same relevance criteria. Sources were also excluded if they were, primarily, based on existing international guidance. A total of 17 guidance reports were selected for appraisal by the authors (Figure 1).

A narrative approach was taken. The author group reviewed the clinical evidence for the use of objective tests to diagnose asthma in children across the identified guidance reports and concluded (by consensus through discussion, in person and virtually) on the clinical use of the following: (1) spirometry; (2) bronchodilator reversibility; (3) peak expiratory flow; (4) bronchial hyper-responsiveness tests; and (5) fractional exhaled nitric oxide. The clinical evidence was then compared with the diagnostic criteria for asthma used in previously conducted asthma prevention trials in children; these trials were identified through a separate targeted literature search, and a selection of examples were included in this review for illustrative purposes.

## 3 Review of clinical recommendations for the diagnosis of paediatric asthma from clinical guidelines

According to GINA, asthma is a heterogenous disease with symptoms that vary over time and in intensity (8). This heterogeneity is one of the major reasons why there are no uniform diagnostic criteria for asthma and, subsequently, why it is not possible to make unequivocal evidence-based recommendations for diagnosing asthma in clinical practice (21, 22). Various national and international initiatives, guidelines, and consensus reports provide recommendations for diagnosing asthma in children (summarised in Table 1). Whilst there are similarities between these sources, a substantial amount of variation exists, highlighting the need for standardised diagnostic criteria for paediatric asthma. This section reviews and evaluates these clinical recommendations.

**Table 1:** Recommendations from international and national clinical guidance reports for the diagnosis of paediatric asthma.

Source	Age (years)	Combination of respiratory symptoms	Clinical assessment^a^	Treatment response^b^	SPT	Allergen-specific IgE	FeNO test	Lung function tests	Other tests
International									
GINA (8)	6–11	Wheeze, shortness of breath, chest tightness, and cough, which vary over time and in intensity, together with variable expiratory airflow limitation	✓	✓	✓	✓	Usefulness not established	Confirmed variable expiratory airflow limitation: • Reduced FEV_1_/FVC (<90%) when FEV_1_ is reduced • Average daily diurnal PEF variability >13% • Variation in FEV_1_ of >12%, or in PEF of >15%	BDR test (salbutamol or equivalent): • Increase of >12% predicted in FEV_1_ Exercise challenge test: • Reduction of >12% predicted in FEV_1_, or >15% in PEF
ICON (23)	5–12	Wheeze, cough, difficulty in breathing, chest tightness triggered by exposure to irritants (e.g., cold, tobacco smoke), allergens (e.g., pets, pollens), respiratory infection, exercise, crying, or laughter	✓	✓	✓	✓	May be a useful tool	Spirometry: • FEV_1_: 80% of predicted, reversible by ≥12% after BDR, 200 ml, or ≥10% of predicted; to be re-assessed PEF (in children able to perform the test): • Wide normal range; more useful for monitoring than diagnosis	BHR: • Provocation with inhaled methacholine, histamine, mannitol, hypertonic saline, or cold air, or exercise Lacking accuracy/standardisation
PRACTALL (24)	≥5	Frequency and severity of wheeze, nocturnal cough, exercise-induced symptoms, and persistence of cough with colds	✓	✓	✓	✓	Further evidence of usefulness required	Spirometry PEF and forced expiratory techniques BDR test (β-agonist reversibility): • Increase of >12% in FEV_1_	Chest x-ray, eosinophil counting in induced sputum and peripheral blood, and basophil histamine release Indirect BHR: provocation with methacholine, histamine, mannitol, hypertonic saline, hyperventilation/cold air, and exercise (preferably running) tests
Europe									
ERS (25)	5–16	Wheeze, cough, and breathing difficulty, together with reversible airways obstruction, airway inflammation, and BHR	✓	✓	NR	NR	FeNO ≥25 ppb plus asthma symptoms supports diagnosis FeNO <25 ppb does not exclude asthma	Spirometry: • FEV_1_/FVC <LLN, and/or <80% • FEV_1_ <LLN, and/or <80% predicted LLN values are derived from the GLI (reference standard for cut-off values) BDR test (with short-acting β_2_-agonist, if positive spirometry): • Increase of ≥12% in FEV_1_	Direct bronchial challenge test (methacholine): • Reduction of 20% in FEV_1_ Indirect bronchial challenge test (bicycle in children with exercise-related symptoms): • Reduction of 10% in FEV_1_
BTS/SIGN (22)	≥5	More than one episode of wheeze, breathlessness, chest tightness, and cough	✓	✓	NR	NR	Positive: ≥35 ppb A negative test does not exclude asthma	Spirometry: • FEV_1_/FVC ratio <90% or <LLN (derived from the GLI) BDR test (with β_2_-agonists or corticosteroids): • Increase of ≥12% in FEV_1_ Compare outcomes during symptomatic and asymptomatic periods, to aid diagnosis	BHR and airway inflammation: • Mannitol challenge • Exercise challenge Blood eosinophilia ≥4%
GEMA (26)	NS	Wheezing (key symptom), dyspnoea, cough, and chest tightness	✓	✓	✓	✓	In the presence of symptoms at ≥6 weeks: >35 ppb (<12 years) or >50 ppb (≥12 years)	Age ≥5 years only Airway obstruction: • FEV_1_/FVC ratio <80%–85% BDR test: • Increase of ≥12% in FEV_1_	Methacholine and exercise challenge tests
NICE (27)	5–16	Wheeze, cough, breathlessness; daily or seasonal variation in these symptoms	✓	✓	NR	NR	Perform if spirometry and BDR are not conclusive Positive: ≥35 ppb	Obstructive spirometry: • FEV_1_/FVC <70% (<LLN if available) BDR test (if positive spirometry): • Increase of ≥12% in FEV_1_ Peak flow variability >20% (if FeNO not conclusive)	NS
Rest of the world									
Asthma + Respiratory Foundation NZ (28)	≤11	Wheeze (most sensitive/specific symptom), breathlessness, chest tightness, and cough	✓	✓ (defined by symptoms and need for reliever medication)	NS	NS	NS	Age ≥5 years Spirometry: • ≥12% response to bronchodilator	NS
Australian Asthma Handbook (29)	≤11	Wheeze, shortness of breath, cough, and chest tightness	✓	✓ (defined by spirometry and symptoms)	✓	NS	NR	Age ≥6 years Reversible airflow limitation: • >12% increase in FEV_1_	Bronchial provocation if diagnosis uncertain
CTS (30)	≥6	Paroxysmal or persistent symptoms, such as dyspnoea, chest tightness, wheezing, sputum production, and cough	✓	✓	NS	NS	NR	Spirometry showing reversible airflow obstruction: • FEV_1_/FVC <LLN (<80%–90%) • Increase of ≥12% in FEV_1_ after bronchodilator treatment or controller therapy Peak expiratory flow variability: • Increase of ≥20% after bronchodilator or controller therapy	Positive challenge test (methacholine challenge): • PC_20_ <4 mg/ml or PD_20_ <0.5 μmol (100 μg) Exercise challenge: • Decrease of ≥10%–15% in FEV_1_
Indian Academy of Pediatrics (31)	NS	Recurrent episodes of wheezing, breathlessness, chest tightness, and cough	✓	✓ (good response: PEF >80% and no symptoms)	NS	NS	NS	Reversible airflow obstruction through PEF Spirometry is optional	NS
Japanese guidelines for childhood asthma (32)	0–15	Dyspnoea with recurrent paroxysmal wheezing and cough	✓	✓	✓	✓	If possible Positive: >35 ppb	Flow volume curve: • FEV_1_/FVC <80%; FEV_1_ <80%; V50, V25 decrease Reversibility test: • Increase of >12% in FEV_1_ PEF monitoring: • Diurnal variation >20%	Exercise load: • Maximum reduction in FEV_1_ of >15% Peripheral eosinophils >300/μl Sputum eosinophils >5%
Malaysian Consensus Statement (33)	NS	Recurrent episodes of cough, wheeze, and/or dyspnoea	✓	✓ (definition not stated)	✓	NS	NS	Response to bronchodilator in older children: • Increase of >15% in FEV_1_ • Improvement in PEF	Only in atypical cases: chest and sinus x-rays; reflux studies; Mantoux test; immune function studies; sweat electrolytes; bronchoscopy; PFTs
NAEPP (34)	5–11	Episodic symptoms of wheeze, cough (worse at night), difficulty breathing, and chest tightness Symptoms occur or worsen in the presence of exercise, viral infection, inhalant allergens, irritants, changes in weather, laughing, or crying	✓	✓	NR	NR	NR	Spirometry: • Reversibility determined by an increase of >200 ml in FEV_1_ and of ≥12% from baseline after SABA provocation	BHR: provocation with methacholine, histamine, cold air, or an exercise challenge test may be useful where spirometry is normal/close to normal Chest x-ray: to exclude other diagnoses Biomarkers of inflammation: including total and differential cell count and mediator assays in sputum, blood, urine, and exhaled air
SINA (35)	0–12 13–18	Recurrent wheezing, cough, shortness of breath, and chest tightness	✓	✓ (definition not stated)	✓	✓ (0–12 years)	NS	Age ≥5 years: Spirometry to show airway obstruction reversibility after bronchodilator therapy Age 13–18 years: Reversible airflow obstruction: • ≥12% in FEV_1_ and ≥200 ml after bronchodilator treatment PEF variability	Age 13–18 years: Bronchoprovocation to rule out atypical asthma with normal spirometry; therapeutic trial with an ICS and bronchodilator combination
Singapore Ministry of Health (36)	NS	Cough, recurrent wheeze/breathing difficulty, or chest tightness Persistent symptoms after age 3 years	✓	✓ (definition not stated)	✓	NR	✓	PEF: • Diurnal variation of PEF ≥15% Spirometry: • Increase in FEV_1_ ≥12% after bronchodilator treatment	Airway challenge tests (exercise or methacholine or histamine inhalation) Mantoux test; otolaryngological evaluation/CT scan of sinuses; gastroesophageal reflux studies; bronchoscopy; immunological investigations
British Columbia GPAC (37)	1–18	Recurrent episodes of wheezing, cough, difficulty breathing, and chest tightness	✓	✓ (no definition stated)	NS	NS	NS	Age 6–18 years Spirometry: • FEV_1_/FVC <80% with a 12% improvement in FEV_1_ after SABA	Methacholine challenge or an exercise challenge, if spirometry is normal

BDR, bronchodilator reversibility; BHR, bronchial hyper-responsiveness; BTS/SIGN, British Thoracic Society/Scottish Intercollegiate Guidelines Network; CT, computed tomography; CTS, Canadian Thoracic Society; ERS, European Respiratory Society; FeNO, fractional concentration of exhaled nitric oxide; FEV_1_, forced expiratory volume in one second; FVC, forced vital capacity; GEMA, Guía Española para el Manejo del Asma (Spanish Guideline on the Management of Asthma); GINA, Global Initiative for Asthma; GLI, Global Lung Function Initiative; GPAC, Guidelines & Protocols Advisory Committee; ICON, International Consensus On Paediatric Asthma; ICS, inhaled corticosteroid; IgE, immunoglobulin type E; LLN, lower limit of normal; NAEPP, National Asthma Education and Prevention Programme; NICE, National Institute for Health and Care Excellence; NR, not recommended; NS, not stated; NZ, New Zealand; PC_20_, provocative concentration that causes a 20% drop in FEV_1_; PD_20_, provocative dose that causes a 20% drop in FEV_1_; PEF, peak expiratory flow; PFT, pulmonary function test; ppb, parts per billion; SABA, short-acting beta agonist; SINA, Saudi Initiative for Asthma; SPT, skin prick test; V25, flow rate at 25% FVC; V50, flow rate at 50% FVC.

^a^ Includes patient/family history and physical examination.

^b^ Bronchodilator and/or anti-inflammatory treatment (unless otherwise stated, a positive result is defined as an increase of ≥12% from baseline in FEV_1_).

### 3.1 Current approach to diagnosing asthma in children

Existing clinical guidance applies mostly to children aged ≥5 years old and recommends performing a clinical assessment (respiratory symptoms, patient/family history, physical examination) and pre- and post-bronchodilator spirometry [to evaluate bronchodilator reversibility (BDR)], as part of the diagnostic work-up for asthma (8, 22–37). Generally, the various guidelines recommend diagnosing asthma through combinations of respiratory symptoms (e.g., wheeze, cough, dyspnoea/shortness of breath, chest tightening) (8, 22–37). Characteristic symptom patterns include the presence of more than one respiratory symptom, symptoms that are often worse at night or early in the morning, and which vary over time and in intensity (8, 24, 26–37). In children, respiratory symptoms can be triggered by respiratory viral infections, exposure to cold, aeroallergens, pollution, and physical activity (among other factors) (8, 23, 24, 26, 28, 29, 34, 35). Objective tests to evaluate lung function or airway inflammation are used to support a diagnosis of asthma (Table 2); these tests include spirometry, peak flow variability, bronchial hyper-responsiveness (BHR; direct and indirect tests), and fractional concentration of exhaled nitric oxide (FeNO) (8, 22–37). The recommended objective test differs depending on the source of clinical guidance (8, 22–37). Ideally, objective testing would be performed before initiating inhaled corticosteroid (ICS) treatment to avoid influencing the results, but the clinical guidance is variable (8, 22–37).

**Table 2:** Summary of objective tests for diagnosing asthma in school-age children as recommended in clinical guidelines.

Test(s)	Benefits	Drawbacks
Spirometry and BDR	• Can demonstrate obstruction and assess reversibility in patients • Essential objective measure as part of the evidence to establish the diagnosis of asthma • Recognised diagnostic tool in paediatric asthma; test and interpretation of result are well standardised (38) • Demonstration of reversible spirometry is practical in large clinical trials due to its simplicity, and standardised method/interpretation	• Guidelines prefer cut-off levels that have high specificity, with the cost of lower sensitivity such that some patients with asthma may remain undiagnosed. The 12% improvement in FEV_1_ is a cut-off with high specificity in relation to asthma, but lacks sensitivity (39) • A positive BDR test (FEV_1_ ≥12%) is not an absolute requirement for diagnosis—some asthmatic children have normal FEV_1_ values and no reversibility (39)
Variability of PEF or FEV_1_	• May be a valuable tool in monitoring asthma in children • Home spirometry with electronic data storage can provide a reliable assessment of asthma (40) • In clinical trials, this could be a means to obtain high-quality longitudinal lung function data	• Wide variability in peak flow meters and reference values. Peak flow meters are designed for monitoring, not as diagnostic tools (34) • Validity has, often, been questioned due to limited reliability of lung function testing in home settings, and wide diurnal variability, particularly in children • Some guidelines consider PEF variation to have a limited role in the diagnosis of asthma in children, but it can be of value if used appropriately
Direct method of measuring BHR (methacholine)	• Very high sensitivity	• Has not been tested in many paediatric studies • Difficult to define normal values in children • Low specificity • Quantitative assessment of BHR with methacholine is considered more useful in ruling out asthma rather than to confirm disease; there are also no standardised cut-off levels to diagnose asthma in children, and BHR tests can be an unpleasant experience for the child • Evidence of BHR in individuals with AR, but without asthma, may be predictive of later disease progression to asthma (41)
Indirect methods of measuring BHR (exercise testing, mannitol tests, eucapnic voluntary hyperpnoea)	Highly specific for asthma, and guidelines for the technical performance and interpretation of these tests are available (42) • Indirect BHR tests also allow objective assessment of asthma in children with normal baseline lung function and without BDR • Exercise testing is the most common indirect test in clinical practice for children, but it is not routinely used as an endpoint in clinical trials • The mannitol test is a standardised indirect provocation test, which has been used as a surrogate of asthma in several clinical trials (43)	• The tests have low sensitivity (42) if asthma is not active • Measurement of BHR is often not feasible in large clinical studies • The methods are poorly standardised
FeNO	• In clinical trials, this marker shows the effect on Th2-driven inflammation and, hypothetically, would be suitable for interventions such as AIT	• Markers of airway inflammation do not, necessarily, cover the whole spectrum of asthma • FeNO is a marker of one inflammatory phenotype, and provides useful information for the management of asthma, but cannot be used as a diagnostic criterion for asthma in an unselected patient sample or population (44) • FeNO values usually normalise rapidly with good adherence to ICS therapy

AIT, allergen immunotherapy; AR, allergic rhinitis; BDR, bronchodilator reversibility; BHR, bronchial hyper-responsiveness; FeNO, fractional concentration of exhaled nitric oxide; FEV_1_, forced expiratory volume in one second; ICS, inhaled corticosteroid; PEF, peak expiratory flow; Th2, T helper type 2.

#### 3.1.1 Spirometry to detect airflow limitation

The most frequently used definition for airflow limitation in spirometry is the forced expiratory volume in one second to forced vital capacity (FEV_1_/FVC) ratio; the thresholds used to indicate airflow limitation can differ between clinical guidelines (see Table 1) (8, 22, 25–27, 30, 32, 37). For example, the UK National Institute for Health and Care Excellence (NICE) defines airflow obstruction as an FEV_1_/FVC ratio of <70% (27), whereas the GINA specifies <90% as the threshold (8). The British Thoracic Society/Scottish Intercollegiate Guidelines Network (BTS/SIGN) state that, in young children, the FEV_1_/FVC ratio can be as high as 90% and, therefore, the commonly used fixed value of 70% considerably underestimates airflow limitation (22). The European Respiratory Society (ERS) has championed a shift to using lower limits of normality (LLN) as the reference standard for spirometry to support a diagnosis of asthma; LLN values have been calculated for different age groups by the Global Lung Function Initiative (25, 44). The ERS guidance recommends using a threshold FEV_1_/FVC ratio, which is <LLN or <80% predicted (25). The use of LLN values is also recommended in the guidance from NICE, BTS/SIGN, and the Canadian Thoracic Society (CTS) (21, 27, 30). Although many children from the age of 5 years are able to perform reproducible spirometry if coached by an experienced technician and with visual incentives, according to GINA (8), it is important to note that some children are incapable of performing sufficient spirometry testing in clinical practice (25).

Our consensus is that, despite the challenges of performing spirometry in children, spirometric testing is fundamental to the assessment of asthma, and should use LLN values as the reference standard.

#### 3.1.2 Bronchodilator reversibility

Some clinical guidelines recommend that, where spirometry is suggestive of asthma, a BDR test should be performed to confirm the diagnosis—generally, an increase in FEV_1_ of ≥12% is considered indicative of asthma (8, 25–29, 35, 37). However, there is considerable variation in normal FEV_1_ between different children meaning that a value in the normal range is not conclusive of normality for individual children. The latest ERS technical standard on interpreting spirometry states an increase of ≥10% of the predicted FEV_1_ as a threshold for a positive bronchodilator response (45). Spanish guidelines state that an 8% increase in FEV_1_ may better define the bronchodilator response in children (26). Guidelines from Malaysia recommend a 15% increase (older children) (33), whereas an increase of ≥12% is recommended in Singapore (36).

Our consensus is that a BDR test should be performed in children, regardless of FEV_1_ and the FEV_1_/FVC ratio. BDR tests should be repeated during symptomatic periods to establish a bronchodilator response (which may also be useful for differential diagnosis), since airway obstruction can be limited when the child is asymptomatic (22, 25).

#### 3.1.3 Peak expiratory flow variability

Measuring peak expiratory flow (PEF) variability is recommended as a supportive objective test for children in some clinical guidelines (8, 23, 24, 27, 31–33, 35, 36) but not in others (22, 25, 26, 28, 29, 34, 35, 37), perhaps because it is less reliable than the alternative measure, FEV_1_ (which is measured in controlled settings) (8). The National Asthma Education and Prevention Program (NAEPP) Expert Panel Report recommends that peak flow meters function better as tools for asthma monitoring, rather than for diagnosis (34). In contrast, the NICE guidelines recommend measuring PEF variability for 2–4 weeks in children, where there is diagnostic uncertainty following an initial assessment (27). The recommended threshold for PEF variability differs between clinical guidelines (see Table 1).

Our consensus is that, for diagnostic purposes, only a PEF variability of ≥20% should be considered suggestive of asthma, in children who are able to perform repeatable tests.

#### 3.1.4 Bronchial hyper-responsiveness tests

Many clinical guidelines recommend BHR tests as a supportive objective diagnostic test for asthma in children (8, 22–27, 29, 30, 32, 34–37). Direct BHR tests involve challenging with methacholine (or histamine), which interacts directly with muscarinic receptors on airway smooth muscle, resulting in contraction and airway narrowing. Indirect BHR tests, such as an exercise or mannitol challenge, elicit bronchoconstriction indirectly through pathways that trigger the narrowing of airways (42, 46). Although such measures can provide valuable supportive evidence (e.g., to establish asthma severity), there is a lack of clarity on the clinical definition of BHR in children. BHR is a hallmark of asthma, but it is a dynamic property—the presence and severity of BHR varies over time, influenced by disease activity, certain triggers, and treatment (47, 48). Furthermore, the quantitative assessment of methacholine responsiveness may be affected by technical factors in the administration of the test substance (46), and by dose–response characteristics that depend on the patient's body size (49). In adults, the association between BHR and asthma is quite strong, but the situation is less clear for children (50). Not all children with recurrent episodes of wheezing have increased BHR, and some children who do not have respiratory symptoms show signs of BHR (50, 51). Furthermore, recent data suggest that, in children with allergic sensitisation, BHR often appears after the onset of respiratory symptoms (52). Most clinical recommendations/guidelines endorse exercise challenge tests as an indirect method of assessing BHR (8, 22–26, 30, 34, 36, 37). Such physiological tests reflect the real-life clinical impact of asthma on children, confirming the relevance of these tests in the clinical evaluation of asthma. However, given that exercise-induced bronchospasm responds rapidly to ICS treatment, exercise challenge tests may be less helpful in patients who are currently receiving ICS treatment.

Our consensus is that exercise tests (running test or standardised exercise challenge test) can be valuable diagnostic tools in paediatric asthma.

#### 3.1.5 Fractional exhaled nitric oxide

FeNO is a marker of type 2 inflammation (22). Perhaps the greatest discord between different clinical guidelines is in the use of the FeNO test as part of the diagnostic work-up for asthma. Some guidelines recommend the use of FeNO (22, 25–27, 32), others highlight that it may be a useful tool (23, 36), while some are of the opinion that the usefulness of FeNO is not yet established (8, 24). The ERS guidelines state that FeNO testing is relatively simple, non-invasive, and accepted by children and their caregivers (25). In contrast, FeNO is not recommended in the guidance from the CTS or the National Asthma Council of Australia (29, 30). Where recommended, the threshold value for a positive FeNO test differs between ≥20 parts per billion (ppb) and 35 ppb depending on the guideline (22, 25–27, 32). The values used to define a positive FeNO test can also be dependent on other factors, such as steroid use (22), and the presence of allergies (atopy is significantly associated with higher levels of FeNO (53) or an airway infection (54). Additionally, in healthy children, FeNO is significantly dependent on the individual's body size (55).

Our consensus is that the FeNO test is regarded as providing supportive, but not diagnostic, evidence for asthma in children, particularly those who are sensitised to allergens.

#### 3.1.6 Navigating the challenges of diagnosing paediatric asthma

Existing clinical guidance highlights the challenges associated with diagnosing asthma in children:

1. Symptoms suggestive of asthma can result from several different conditions, which underlines the importance of considering differential diagnoses (8). Other illnesses, such as viral respiratory tract infections, can cause children to wheeze (8), manifesting as transient obstructive lung function that is reversible with a bronchodilator.
2. Significant BDR is often not observed when children are well (regardless of treatment with ICSs) and may be evident in children without asthma (e.g., following lower respiratory tract infections).
3. BHR is observed in children with AR, but without asthma (56), complicating its use as a diagnostic test for asthma in atopic children.
4. Elevated FeNO levels can be observed in children with AR (57), or in asymptomatic children with allergic sensitisations (58).

In clinical practice, algorithms combining a history, or presence of, respiratory signs and symptoms with supportive objective tests, are important in the diagnosis of asthma. No single diagnostic test can appropriately diagnose new-onset paediatric asthma and, therefore, the combination of different tests and patient clinical history must be considered. However, the Swiss Paediatric Airway Cohort study showed that the NICE and GINA algorithms for the diagnosis of paediatric asthma in children aged 5–17 years are challenging to apply in an outpatient setting and did not agree well with the diagnosis made by pulmonologists (59).

#### 3.1.7 Summary of recommendations from clinical guidance

Despite an overall lack of consensus in the published clinical guidance, there are similarities between the various recommendations for diagnosing asthma in children, which typically involve supplementing medical history with objective measures of lung function. Most guidelines agree that no single symptom, sign, or test can be used alone to diagnose paediatric asthma, and that the predictive value of diagnostic tests is influenced by the context. A broader approach to diagnosis, involving a period of observation of the variable symptoms/signs of asthma to confirm or exclude asthma, may be preferable.

## 4 Review of new-onset asthma definitions in paediatric asthma prevention trials

The published European Medicines Agency (EMA) guidance for the development of medications for asthma is focused on the symptomatic treatment of asthma. It does not include standard criteria for diagnosing asthma, or recommendations for primary endpoints to assess the prevention of new-onset asthma in clinical trials (60, 61). Therefore, the guidance may not be directly relevant to clinical trials of treatments designed to prevent new-onset asthma (61). Instead, the guidance states that the diagnostic criteria for asthma in children aged ≥6 years should be based on the recommendations outlined in existing clinical guidelines (61). Consequently, the criteria for diagnosing asthma vary between the numerous clinical trials that have been conducted to evaluate the potential of different treatments in preventing the onset of asthma in children (Table 3) (62–70).

**Table 3:** Definitions of new-onset asthma used in paediatric clinical trials assessing asthma prevention.

Trial identifier	Intervention(s)	Definition of new-onset asthma
Examples of AIT asthma prevention trials		
Preventive allergy treatment (PAT) (62)	Birch and/or grass pollen SCIT Placebo	Asthma was defined as a recurrence of at least two of the following symptoms within the previous 12 months: • Cough • Wheeze • Shortness of breath A conclusive diagnosis of asthma required that symptoms were triggered not only by infections, and that the patients responded to treatment with β_2_-agonists. The clinical diagnosis was based only on the appearance of repeated symptoms and was independent of the level of hyper-responsiveness
Novembre et al. Allergy Clin Immunol (2004) (63)	Co-seasonal SLIT drops from an extract of mixed grass pollens Control group	Asthma was defined as at least three episodes of wheezing/breathing difficulty, cough, or both (separated by at least 1 week) that required bronchodilator therapy for symptom relief, and where conditions other than allergy had been excluded
Grazax Asthma Prevention (GAP) (20)	SQ grass SLIT-tablet (GRAZAX®) Placebo	Asthma was defined as meeting at least one of the following criteria, which were evaluated at each trial visit for each time period “since last visit”: • At least one episode of wheeze, cough, shortness of breath, or chest tightness, and a change in FEV_1_ ≥12% after β_2_-agonist administration • Wheezing with or without prolonged phase of forced exhalation observed at physical examination and an intake of asthma medication, which resulted in a clinically relevant effect • Wheezing with or without prolonged phase of forced exhalation observed at physical examination and a change in FEV_1_ ≥12% after β_2_-agonist administration The onset of asthma was considered a binary event (asthma yes/no) that could occur only once per patient. Therefore, children were classified as having asthma if the criteria were met at a single given visit only; clinical information from previous or subsequent visits was not taken into consideration when this classification was made
Mite allergy prevention study (MAPS) (64)	HDM SLIT drops Control group	For the outcome of asthma, all cases were reviewed by a blinded adjudication committee with expertise in asthma. The information used included symptom history, response to medication, spirometry, reversibility, FeNO, and BHR. Based on this information, a diagnosis of asthma was confirmed or excluded. Participants were allocated to one of five categories: “definite asthma”, “probable asthma”, “possible asthma”, “probable not asthma”, and “definite not asthma”
Examples of non-AIT asthma prevention trials		
Marks et al. J Allergy Clin Immunol (2006) (65)	HDM avoidance intervention Diet supplemented with omega-3 fatty acids Control group	Probable current asthma (at the age of 5 years) defined as parental report of any wheeze in the previous 12 months at age 5 years, and either a parental report of diagnosed asthma at ages 18 months, 3 years, or 5 years, or a >12% increase in FEV_1_ after bronchodilator at age 5 years
Early treatment of the atopic child (ETAC) (66)	Cetirizine oral solution Placebo	Time to the onset of asthma, defined by three episodes separated by at least 7 days of: • Nocturnal cough with sleep disturbance lasting for at least three consecutive nights • Three episodes of wheezing • A combination of the two
COPSAC_2010_ (67)	Vitamin D_3_ (2,400 IU/day) Placebo (Plus 400 IU/day vitamin D_3_ as usual care)	Asthma was diagnosed in children fulfilling the persistent wheeze criteria at age 3 years: • Recurrent wheeze (verified diary recordings of at least five episodes of troublesome lung symptoms (cough, wheeze, and/or dyspnoea) • Typical symptoms of asthma (e.g., exercise-induced symptoms, prolonged nocturnal cough, or persistent cough outside common cold) • Need for intermittent bronchodilator • Response to a 3-month trial of inhaled corticosteroids and relapse upon cessation
Trial of infant probiotic supplementation (TIPS) (68)	Probiotics (10 billion colony-forming units of LGG and 225 mg of inulin) Control group (325 mg inulin)	Asthma was diagnosed based on repeated parental reports of the diagnosis by a blinded clinician, on two occasions. Early markers of asthma included a history of frequent wheezing, wheezing without a cold, atopic dermatitis, and rhinitis (using a standard history assessment). Additional laboratory markers included elevated serum immunoglobulin and eosinophilia
Vitamin D antenatal asthma reduction trial (VDAART) (69)	Vitamin D supplementation Control group	Asthma was defined as a maternal or caregiver report of physician-diagnosed asthma (including wheezing and medication use), and time of onset was defined as the first report of wheezing or the first report of the use of any asthma medication (bronchodilator inhalers/nebulisers, pills, or syrups; or steroid inhalers/nebulisers; or leukotriene modifiers; or steroid pills or liquids)
Preventing asthma in high-risk kids (PARK) (70)	Omalizumab Placebo	Asthma was defined (at the end of the observation period) as having one of the following: • At least one hospitalisation for wheezing/asthma • At least 6 months of asthma controller use • At least two wheezing episodes • At least two doctor or emergency department visits for asthma • FEV_1_ reversibility ≥10% after four puffs of albuterol plus at least one wheezing episode and at least one doctor/emergency department visit for wheezing/asthma A wheezing episode is defined as parental or documented report of an episode of wheezing or whistling in the chest that lasts at least 24 h. Wheezing events separated by at least 5 consecutive days without wheezing shall be counted as separate episodes

AIT, allergen immunotherapy; BHR, bronchial hyper-responsiveness; COPSAC, Copenhagen Prospective Studies on Asthma in Childhood 2010; FeNO, fractional concentration of exhaled nitric oxide; FEV_1_, forced expiratory volume in one second; HDM, house dust mite; ICS, inhaled corticosteroid; LGG, *Lactobacillus rhamnosus* GG; SCIT, subcutaneous immunotherapy; SLIT, sublingual immunotherapy.

Although many of these asthma prevention trials defined new-onset asthma primarily by asthma symptoms and medication use, the variation in criteria highlights the lack of a standard definition for new-onset asthma in paediatric clinical research. This section focuses on selected trials that have investigated AIT in preventing the development of asthma, alongside some examples of non-AIT asthma prevention trials.

### 4.1 Asthma prevention trials with allergen immunotherapy (AIT)

To illustrate how different definitions of asthma onset may impact the results in asthma prevention trials, we describe two large, randomised trials that have assessed the preventive effect of AIT on asthma development.

The Preventive Allergy Treatment (PAT) trial was a randomised open-label trial investigating the preventive effect of AIT on the risk of developing asthma (62). The trial randomised 205 children (aged 6–14 years) with grass or birch pollen AR to either symptom-relieving medication alone or symptom-relieving medication plus SQ subcutaneous immunotherapy (SCIT) (grass and/or birch) (62). Children with asthma requiring daily asthma treatment were excluded (62). Asthma was defined by trial investigators, based on the recurrence of at least two asthma symptoms (cough, wheeze, and/or shortness of breath) and responsiveness to β_2_-agonist treatment (confirmation of the asthma diagnosis by an objective test was not required) (Table 3) (62). Three years of SQ SCIT treatment increased the likelihood that children did not develop asthma vs. children who were not treated with SQ SCIT {odds ratio [OR] = 2.52; [95% confidence intervals (CI): 1.3, 5.1]; *p* < 0.05} (62). This clinical benefit for the prevention of new-onset asthma was observed long term, for up to 7 years after SCIT treatment completion [OR at Year 10 = 2.5 (95% CI: 1.1, 5.9)] (41).

The later GAP trial was conducted to investigate asthma prevention with SQ grass sublingual immunotherapy (SLIT) tablet vs. placebo in children with grass pollen AR, using a blinded randomised controlled trial design (20). The 5-year GAP trial enrolled 812 children (aged 5–12 years) and included a 3-year treatment course followed by a 2-year follow-up period (20). Children with any history of asthma and/or wheezing, or signs of asthma, were excluded (20). The protocol-specific asthma diagnostic criteria were devised during consultation with the EMA. This approach required an objective measure (BDR) in addition to an evaluation of asthma symptoms and medication use, resulting in a more stringent definition of asthma compared to that used in other trials with a similar objective (i.e., to evaluate asthma prevention) (Table 3) (20). No effect of SQ grass SLIT-tablet was observed on the primary endpoint of time to onset of protocol-defined asthma vs. placebo (20). Concerningly, some of the children who met the diagnostic criteria for asthma in the first part of the 3-year treatment period did not show signs of asthma later in the trial, whereas many other children who did not meet the diagnostic criteria during the trial did show signs suggestive of asthma (20). This finding indicated, to the investigators, that the definition of new-onset asthma used in the trial had not performed well—a probable reason being that, in children, a diagnosis of asthma is difficult to make on the basis of a single point-in-time evaluation that relies on demonstrating reversible lung function impairment. Rather, the diagnosis of asthma, in the context of clinical trials, needs to be based on a combined clinical assessment with objective testing conducted over a longer observation period, as is usually performed in routine clinical practice (20, 22, 25, 71, 72). Although a *post hoc* analysis conducted at the end of the 5-year trial period showed a significant effect for the SQ grass SLIT-tablet on reduction of asthma symptoms and asthma medication use (20), this is insufficient to confirm a diagnosis of asthma.

### 4.2 Non-AIT asthma prevention trials

We also examined a few asthma prevention trials that investigated interventions other than AIT. For these trials, the diagnostic criteria were based mainly around clinical symptoms (and also asthma medication use in some trials) and are, therefore, less stringent than the criteria used in the AIT trials (Table 3). Of the non-AIT trials listed in Table 3, none of the interventions showed a beneficial effect on asthma prevention, despite the different asthma diagnostic criteria applied across the trials (65–69). The “Preventing Asthma in High Risk Kids (PARK)” trial is ongoing (70).

### 4.3 Summary of asthma prevention trials

In summary, for clinical trials in asthma (including paediatric AIT and non-AIT trials), the regulatory recommendation is to diagnose asthma according to the clinical guidelines, which are clearly variable; however, the dynamic nature of the disease must also be considered. The paediatric asthma prevention trials conducted highlight the challenges of defining diagnostic criteria for asthma and, consequently, there is a need for standardised criteria to define new-onset asthma in a paediatric clinical trial setting.

## 5 Suggestions for the diagnosis of new-onset asthma in future paediatric clinical trials

In this section, we consider how new-onset asthma could be diagnosed in future clinical trials. The recurring and dynamic nature of asthma creates challenges when diagnosing new-onset asthma, and emphasises the need for regular evaluation over time with standardised objective measurements, as is done in routine clinical practice.

### 5.1 Self-reported symptoms

**Figure 2:**
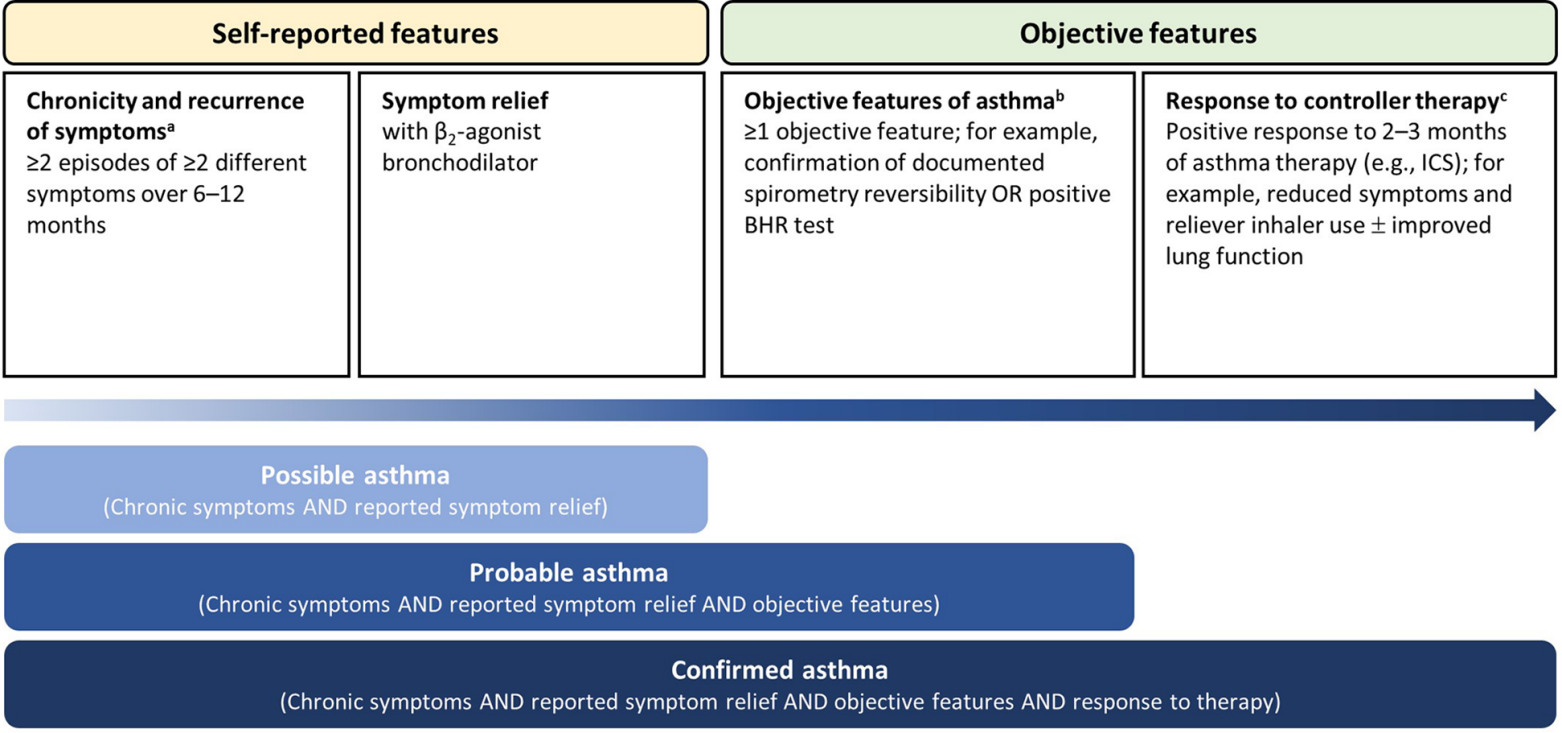
A suggested approach to diagnose new-onset asthma in a paediatric clinical trial (school-age children). ^a^Including wheeze, shortness of breath, chest tightness, cough, exercise-induced symptoms, persistence of cough with colds, and daily or seasonal variation in symptoms. Symptoms may also be triggered by exposure to irritants (cold, tobacco smoke), allergens (pets, pollen, house dust mite, etc.), respiratory infection, exercise, crying, or laughter. Exclude symptoms of alternative diagnosis. ^b^Preferably, objective diagnostic tests should not be performed during treatment with controller medication. ^c^In the case of poor response to controller therapy, a diagnosis of asthma is unlikely if ICS has been administered at an adequate dose with optimal inhalation technique and adherence. BHR, bronchial hyper-responsiveness; ICS, inhaled corticosteroid.

Chronicity and recurrence of symptoms (self-reports using patient-reported outcomes) need to be captured with a follow-up lasting 6–12 months. Patients should have experienced at least two episodes of at least two different characteristic asthma symptoms (including wheeze, shortness of breath, chest tightness, cough, exercise-induced symptoms). When chronic symptoms are documented, patient-reported symptom relief with β_2_-agonist bronchodilator treatment should be evaluated (indicating reversibility). Documented symptom relief in the presence of chronic symptoms is suggestive of “possible” asthma (Figure 2).

### 5.2 Objective features

The Lancet Asthma Commission Report emphasises the importance of performing objective measurements (73). We suggest that objective evidence should be obtained either as spirometry reversibility with an inhaled β_2_-agonist or a positive BHR test (methacholine or exercise test). Measuring BDR is an important clinical tool to monitor the effect of bronchodilators, but also to document reversible airway obstruction. Given that objective tests differ in sensitivity and specificity (Table 2), the diagnostic rate of asthma would vary dependent on the specific criteria used for the objective test. Since the full range of tests may not be available in all centres, the appropriate objective test should be tailored to each trial. Our preference would be for spirometry reversibility in children ≥6 years of age conducted before initiation of ICS therapy. Data from objective testing undertaken by other healthcare professionals should also be assessed as part of the trial protocol to minimise the chance that ICS treatment has already been initiated before the trial assessment and reversibility was missed. A BHR test may be a helpful supplementary objective marker to define asthma (42), even during periods of normal baseline lung function.

### 5.3 A suggested approach in school-age children

In a clinical trial setting, it is important to allow for unscheduled visits and to recall children when they are symptomatic to maximise the chance of capturing data when they have objective features and chronic symptoms. In cases where chronic symptoms are documented, patient-reported symptom relief with a β_2_-agonist bronchodilator has been reported (indicating reversibility), and there is evidence of positive objective tests (e.g., documented spirometry reversibility), we suggest that a diagnosis of “probable” asthma can be made (Figure 2). We also suggest 2–3 months of controller medication treatment with an ICS (Figure 2), and to reassess the treatment response through clinical assessment and objective measures, such as lung function, to confirm an asthma diagnosis. A positive response may include a reduction in reported symptoms and in the use of reliever inhaler, as well as improved lung function (on objective measures, such as spirometry and reversibility); this would deliver a conclusion of “confirmed” asthma (Figure 2). If the response to asthma therapy is poor, correct inhaler technique and adherence to the medication should be checked; further testing should be arranged, and alternative diagnoses considered.

## 6 Discussion

Review of the published clinical guidelines emphasises the importance of a broader approach to the diagnosis of asthma in children, including observation of the variable symptoms/signs of asthma over time supplemented with objective measures of lung function. Although a full consensus across clinical guidelines on the use and thresholds of different lung function tests is lacking, most guidelines agree that the predictive value of diagnostic tests is influenced by the context, and that no single test is sufficient to diagnose paediatric asthma alone.

The BTS/SIGN guidance acknowledges that asthma status and the outcome of diagnostic tests can vary over time, meaning that objective tests performed when patients are asymptomatic or during an “inactive period” (i.e., not having received a prescription for a year) may produce false negative results (22). Likewise, the PRACTALL consensus report highlights that, often, a diagnosis of asthma in children is possible only by understanding the symptom patterns over an extended period of time, observing a child's response to bronchodilator and/or anti-inflammatory treatment at different times (24).

It is important to ensure that the objective test of choice to confirm a diagnosis of asthma is appropriate for the age of the child. Objective tests are useful only when they are consistently performed properly and, therefore, should not be used for diagnosing asthma without evaluation of a patient's technique. Furthermore, it is important to remember that a normal result on any given objective test does not exclude asthma (22). Airway obstruction can be limited when the patient is asymptomatic or in the early onset of asthma symptoms (22, 25). Therefore, it is important to consider the optimal timing and frequency of objective tests in diagnosing new-onset asthma. Given that different centres employ different tests and that some tests are not appropriate for all age groups, an element of heterogeneity will be introduced into the definition due to the differences in sensitivity and specificity between the tests (Table 2). However, the inclusion of an objective component at the correct time in the trial pathway should improve diagnostic accuracy compared with the current situation.

Recent evidence-based guidance on defining new-onset asthma from clinical guidelines has not been implemented into clinical trial protocols, which has been to their detriment. Review of published paediatric asthma prevention trials showed that many different diagnostic criteria for new-onset asthma have been applied, and that many trials were focussed, primarily, on asthma symptoms and medication use. The variation in asthma diagnostic criteria highlights the lack of a standard definition for new-onset asthma in paediatric clinical research.

From asthma prevention trials using AIT and other interventions, we have learned that evaluation of chronic symptoms is important to establish a diagnosis of new-onset asthma. An assessment of asthma onset at one single timepoint can lead to both under- and over-diagnosis (6). Asthma is a dynamic condition. When diagnosing asthma, it is important to evaluate the chronicity and persistence of more than one clinical symptom, perform one or more objective tests, and monitor the clinical response to asthma treatment. We have made suggestions for diagnosing new-onset asthma in paediatric clinical trials, which encompass these key considerations. The suggestions aim to improve the accuracy of diagnosing asthma in school-age children, and the design and results of future clinical trials. In the absence of agreed standardised worldwide diagnostic criteria for asthma in children, we hope that our suggestions bring the definition of new-onset asthma used in clinical trials in line with current evidence-based recommendations and, in doing so, we may help to diagnose new-onset asthma in future paediatric asthma prevention trials.
